# Synthesis and Study of the Effect of Ba^2+^ Cations Substitution with Sr^2+^ Cations on Structural and Optical Properties of Ba_2−*x*_Sr*_x_*ZnWO_6_ Double Perovskite Oxides (*x* = 0.00, 0.25, 0.50, 0.75, 1.00)

**DOI:** 10.3390/ma10050469

**Published:** 2017-04-28

**Authors:** Yousef A. Alsabah, Mohamad S. AlSalhi, Abdelrahman A. Elbadawi, Eltayeb M. Mustafa

**Affiliations:** 1 Department of Physics, Faculty of Science and Technology, Al Neelain University, Khartoum 13314, Sudan; y.a.alsabah@gmail.com (Y.A.A.); bahlaoy.ab@gmail.com (A.A.E.); eltayebmmustafa@hotmel.com (E.M.M.); 2 Research Chair in Laser Diagnosis of Cancers, Department of Physics and Astronomy, College of Science, King Saud University, Riyadh 11451, Saudi Arabia; 3 Department of Physics, Faculty of Education and Applied Science, Hajjah University, Hajjah 1729, Yemen

**Keywords:** double perovskite, crystal structure, morphology, molecular structure, UV-visible, diffuse reflectance

## Abstract

The effect of Sr^2+^ substitution on the morphology, crystal structure, and optical properties of double perovskite oxide Ba_2−*x*_Sr*_x_*ZnWO_6_ (*x* = 0.00, 0.25, 0.50, 0.75, 1.00) were investigated. Scanning electronic microscopy demonstrated that all samples have similar microstructure morphology but differ in the range of grain sizes. X-ray diffraction measurements indicated that these materials crystallize in a (Fm-3m) cubic crystal structure, and also confirmed the tolerance factor. Rietveld analysis revealed that the lattice parameter decreased from 8.11834 to 8.039361 Å when the substitution of Ba^2+^ with Sr^2+^ cations increased from zero to 100%. Fourier transform infrared (FTIR) and Raman spectroscopies displayed a symmetric stretching vibration of WO_6_ octahedra at 825 cm^−1^, and an anti-symmetric stretching mode of WO_6_ was observed by FTIR at 620 cm^−1^. A strong peak at 420 cm^−1^ was also observed in the Raman spectra and is due to the W–O–W bending vibration modes. UV-Vis diffuse reflectance spectroscopy was carried out for the series, and the band gap energy decreased from 3.27 eV for Ba_2_ZnWO_6_ to 3.02 and 3.06 eV for Ba_1.75_Sr_0.25_ZnWO_6_ and Ba_1.5_Sr_0.5_ZnWO_6_, respectively. The excitation and emission photoluminescence properties were investigated at room temperature.

## 1. Introduction

Many researchers are interested in double perovskite oxides that consist of transition metals [1]. These materials represent a large part of material science research because of the diversity in their physical and chemical characteristics, and their diverse applications [1,2,3,4,5,6], such as light harvesting (LaNiMnO_6_) [2], ferroelectrics (Pb_2_Mn_0.6_Co_0.4_WO_6_) [5], Multiferroic (Bi_2_NiMnO_6_, Bi_2_FeCrO_6_) [6], superconductivity (Sr_2_YRu_0.95_Cu_0.05_O_6_) [7], magneto resistance (Sr_2_FeMoO_6_) [8], dielectric resonators (Ca_2_AlTaO_6_, SrAlTaO_6_) [9], and photo-catalysis (Cs_2_BiAgCl_6_) [10]. 

A variety of devices and methods have previously been used to find and characterize new double perovskite compounds at high temperatures. Zaraq et al. [1] synthesized SrCaCoTeO_6_ and SrCaNiTeO_6_ compounds and used X-ray diffraction (XRD) and scanning electron microscopy (SEM) to describe their crystal structure and phase transition. Chufeng Lau et al. [2] used XRD, photoelectron spectroscopy, and UV-Vis-NIR spectroscopy to describe the LaNiMnO_6_ compound and studied the possibility for its solar cell applications. Paiva et al. [3] used the PANalytical diffractometer and Solartron 1260 impedance analyzer to study the structure and microwave properties of Sr_3_WO_6_, which is used in Bluetooth and mobile system devices for microwave telecommunications through wireless antennae. By utilizing the conventional solid-state ceramic route, BiCu_2_VO_6_ and BiCa_2_VO_6_ powders, which are used in low-temperature co-fired ceramic applications, were prepared and examined using XRD, SEM, and the TE_01δ_ shielded cavity method with a network analyzer (8720ES) and temperature chamber (Delta 9023) to characterize their structure and microwave dielectric behaviors. Orlandi et al. [5] utilized the solid-state reaction route to synthesize Pb_2_Mn_0.6_Co_0.4_WO_6_, and used XRD with the SQUID MPMS Quantum Design magnetometer in order to investigate its crystal and magnetic structure. In addition, the perovskite compound can be used in biomedical applications. LaNiMnO_6_ nanoparticles were synthesized using the co-precipitation method and were characterized by XRD using a vibration magnetometer (PPMS-9, Quantum design), Transmission electron microscopy (TEM), and UV-VIS spectroscopy to investigate the structure, magnetic, and adsorption of bovine serum albumin applications. The nanoparticles displayed good adsorption performance in the bovine serum albumin proteins. The Double-perovskite La_2_NiMnO_6_ (LNMO) nanoparticles are potential carriers for large biomolecules, which have wide use in biomedical applications.

The general chemical formula of double perovskite oxide is expressed as AA′BB′O_6_, and the crystal structure of AA′BB′O_6_ consists of the exchange sites of BO_6_ and B′O_6_ octahedra across the corners of the network connection. The A and A′ atoms exist in the space between the BO_6_ and B′O_6_ octahedra, and can be any element from groups 1 and 2 of the periodic table, especially rare earth elements, while the B and B′ cations can be any transition element [1,11]. 

The double-perovskite oxide compounds have a very high flexibility in crystal structure and chemical composition, where it is possible to vaccinate or replace the A-sites and B-sites cations with the continuation of the octahedra network connection [1,11] such as Sr_2_FeMo_1−*x*_W*_x_*O_6_ (where 0 ≤ *x* ≤ 1) [12], Ba_2_Mg_1−*x*_Ca*_x_*WO_6_ (where 0.0 ≤ *x* ≤ 0.15) [13], Ca_3_WO_6_:Dy^3+^ [14], Sr_2_MWO_6_ (where M = Co, Ni) [15], and Sr_2_Ca_1−2*x*_Eu*_x_*Na*_x_*MoO_6_ [16]. In this study, we use XRD, SEM, Fourier transform infrared (FTIR) spectroscopy, photoluminescence, and UV-Vis diffuse reflectance to study the structure and optical properties of the Ba_2−*x*_Sr*_x_*ZnWO_6_ double perovskite series (*x* = 0.00, 0.25, 0.50, 0.75, 1.00) and discuss the effect of Ba^2+^ cation substitution with Sr^2+^ cations in series behavior.

## 2. Results and Discussion

### 2.1. Structural Characterization

#### 2.1.1. Scanning Electron Microscopy

**Figure 1:**
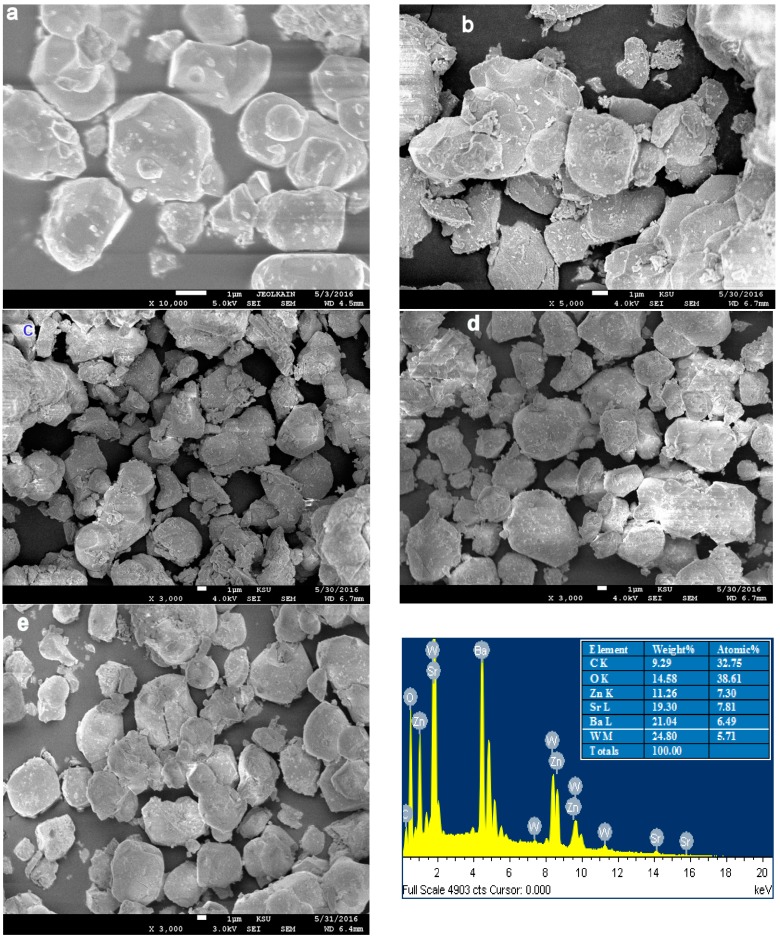
(**a**) SEM image of Ba_2_ZnWO_6_; (**b**) SEM image of Ba_1.75_Sr_0.25_ZnWO_6_; (**c**) SEM image of Ba_1.5_Sr_0.5_ZnWO_6_; (**d**) SEM image of Ba_1.25_Sr_0.75_ZnWO_6_; (**e**) SEM image and EDX spectroscopy of BaSrZnWO_6_.

The ESM images of the Ba_2−*x*_Sr*_x_*ZnWO_6_ (*x* = 0.00, 0.25, 0.50, 0.75, 1.00) series are displayed in Figure 1a–e. The morphologies of all samples are identical and they appear to be highly homogeneous with no impurities. It is observed that, in all samples, the size of the particles is large and they are aggregated in groups, which is due to the higher preparation temperature. Chunfeng Lan et al. [2] observed an equivalent effect of temperature in the morphology of La_2_NiMnO_6_ double perovskite oxide. Furthermore, the images reveal the presence of fine fragments that are produced during the preparation grinding. Each of the samples has grains of various sizes, i.e., Ba_2_ZnWO_6_ has 1–3 μm grains, Ba_1.75_Sr_0.25_ZnWO_6_ has 1–5 μm grains, Ba_1.5_Sr_0.5_ZnWO_6_ has 1.5–4 μm grains, Ba_1.25_Sr_0.75_ZnWO_6_ has 2–8 μm grains, and BaSrZnWO_6_ has 2–7 μm grains. An Energy-dispersive X-ray spectroscopy (EDX) analysis is conducted with each sample using the SEM images. Figure 1e presents the energy dispersive X-ray spectrum from the element that formed the BaSrZnWO_6_ sample. All EDX graphs confirm that all samples contain elements of the raw material preparation composition and a proportion approximating the input quantities to configure each sample with a small error, which refers to the homogeneity and crystal purity.

#### 2.1.2. X-ray Powder Diffraction

**Figure 2:**
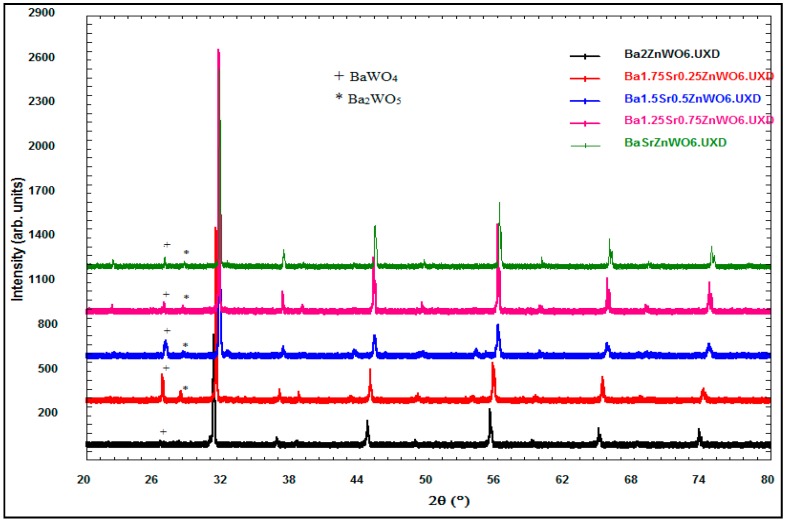
X-ray powder diffraction of Ba_2−*x*_Sr*_x_*ZnWO_6_ series.

**Figure 3:**
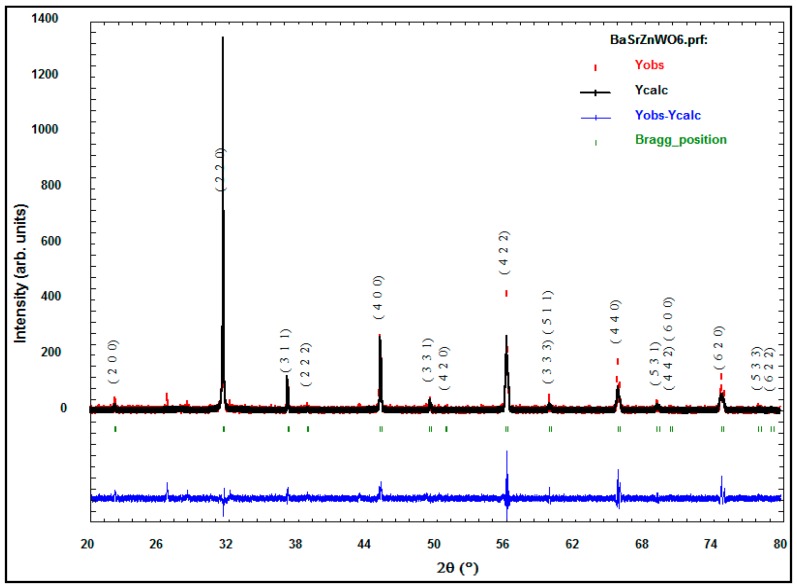
Refined XRD patterns of the BaSrZnWO_6_.

The X-ray diffraction data of the perovskite oxide compounds are essential in determining the crystalline structure of the samples in terms of the Bravais lattice, atomic position, lattice parameter, and space group. Many studies refer to the importance of the study of material structure since they govern the other properties of the materials [17]. The XRD of the Ba_2−*x*_Sr*_x_*ZnWO_6_ (*x* = 0.00, 0.25, 0.50, 0.75, 1.00) double perovskite oxide series prepared by the solid-state reaction is shown in Figure 2. The BaWO_4_ and Ba_2_WO_5_ phases displayed as minor peaks at low intensity in the XRD pattern shown in Figure 1 are attributed to the impurities in the Ba_2−*x*_Sr*_x_*ZnWO_6_ (*x* = 0.00, 0.25, 0.50, 0.75, 1.00) structure around 26° and 28° [18]. A plus sign and a star are used to depict them in Figure 1 when 2θ is around 26° and 28° for BaWO_4_ and Ba_2_WO_5_, respectively. The XRD data of each sample in the series are refined by the Rietveld method using the FullProf program. Table 1 displays the atom coordinates of all the samples obtained in the (Fm-3m) cubic crystal structure. Figure 3 shows the XRD refinement of BaSrZnWO_6_, which is represented by a (Fm-3m) cubic structure with lattice parameters *a* = *b* = *c* = 8.039361 Å and *α* = *β* = *γ* = 90°. Identical results are obtained for Ba_2_ZnWO_6_ using single-crystal X-ray and neutron diffraction [19]. Furthermore, the Ba_2−_*_x_*Sr*_x_*MgTeO_6_ series was found in a (Fm-3m) cubic crystal structure [20]. The crystallite size was calculated from the Full width at half maximum (FWHMs) at the major peaks at (220) for the Ba_2−*x*_Sr*_x_*ZnWO_6_ (*x* = 0.00, 0.25, 0.50, 0.75, 1.00) double perovskite series using the Scherer equation [21], which was observed to vary between 47.41 and 105.9 nm for the samples.

(1) $$D=\frac{0.94\;\lambda }{\beta \cos\theta },$$

where *D* is the crystalline size, *λ* is the wavelength (1.5405 Å), *β* is the full width at half maximum, and *θ* is the diffraction angle. The tolerance factor was found to be between 0.998 and 1.007, which can be calculated by

(2) $$t=\frac{\left(1-\left(\frac{x}{2}\right)r_{A^{`}}\right)+\frac{x}{2}r_{A^{``}}+r_{o}}{\sqrt{2\;}\;\left(\frac{r_{B^{`}}}{2}+\frac{r_{B^{``}}}{2}+r_{o}\right)}$$

Table 2 displays the tolerance factor and parameter of the crystal structure of Ba_2−_*_x_*Sr*_x_*ZnWO_6_ (*x* = 0.00, 0.25, 0.50, 0.75, 1.00) using the Rietveld method of refinement. The unit cell volume decreases with an increasing substitution as a result of the larger ionic radius of the Ba^2+^ cation than that of Sr^2+^.

### 2.2. Optical Studies

#### 2.2.1. FTIR Spectroscopy

**Figure 4:**
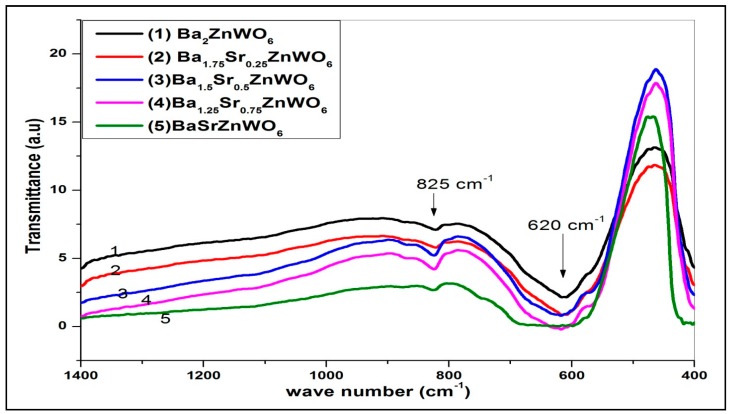
The Fourier transform infrared (FTIR) spectra of the Ba_2−*x*_Sr*_x_*ZnWO_6_ double perovskite series.

The FTIR spectra identify the crystal structure of the perovskite structure materials that have characteristic absorption bands in the 850–400 cm^−1^ region [22]. The strong high-energy anti-symmetric stretching mode of the WO_6_ octahedral displayed at 620 cm^−1^ is due to the higher charge of the tungsten cations. The symmetric stretching vibration of the WO_6_ octahedral appears as a high-intensity band at about 825 cm^−1^. Figure 4 shows the transmittance of the Ba_2−*x*_Sr*_x_*ZnWO_6_ double perovskite series versus wave number, and all the samples confirm the molecular bands on the perovskite oxide structure [17].

#### 2.2.2. Raman Spectroscopy

**Figure 5:**
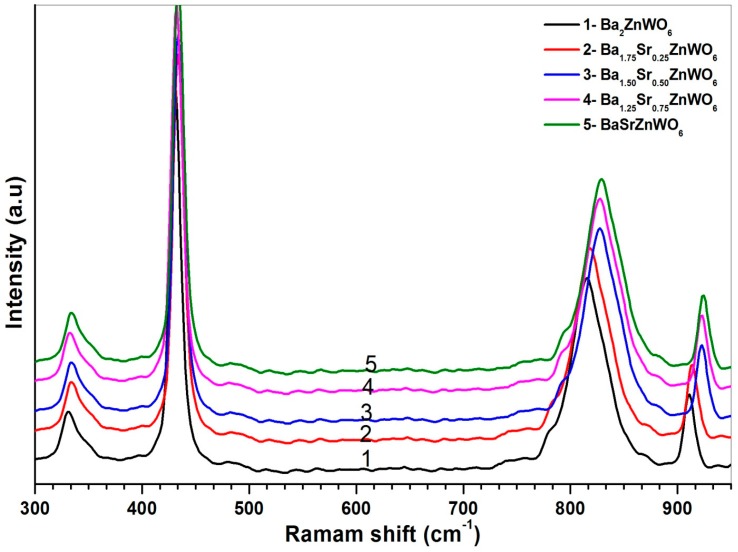
The Raman shift spectra of the Ba_2−*x*_Sr*_x_*ZnWO_6_ double perovskite series.

The Raman spectra of the samples are shown in Figure 5 for Ba_2−*x*_Sr*_x_*ZnWO_2_ (*x* = 0.00, 0.25, 0.50, 0.75, 1.00). For all samples, the Raman modes are classified into two types of lattice vibration—the W–O–W bending vibration in the 200–500 cm^−1^ region and the W–O stretching mode between 700 and 950 cm^−1^. This result was observed in many studies on double perovskite oxides [22,23]. Ba has a larger ionic radius (149 Å) than that of Sr (132 Å). When the Ba substitution increases, the effect of the Ba radius is reflected by a decrease in both the bending and stretching modes of the W–O bonds. A redshift in the Raman energy is also observed.

#### 2.2.3. UV-Visible Diffuse Reflectance Spectroscopy

**Figure 6:**
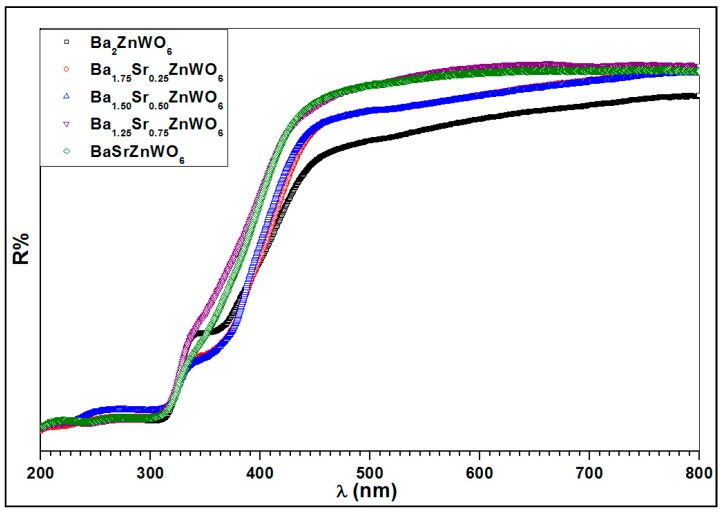
The diffuse reflection spectrum for Ba_2−_*_x_*Sr*_x_*ZnWO_6_ (*x* = 0.00, 0.25, 0.5, 0.75, and 1.00) double perovskite series.

Figure 6 presents the diffuse reflectance spectrum of the Ba_2−*x*_Sr*_x_*ZnWO_6_ series at room temperature in the 200–800 nm range. The strong absorption band observed at 300–450 nm refers to the absorption edge in tungsten due to the charge transfer transition of $W^{6+}-O^{2-}$ in the lattice from the highest filled molecular orbital 2p of oxygen to the lowest empty molecular orbital 5d of tungsten [14,19,24]. The absorption band has a blue shift with an increasing substitution ratio of Ba^2+^ with Sr^2+^ cations.

**Figure 7:**
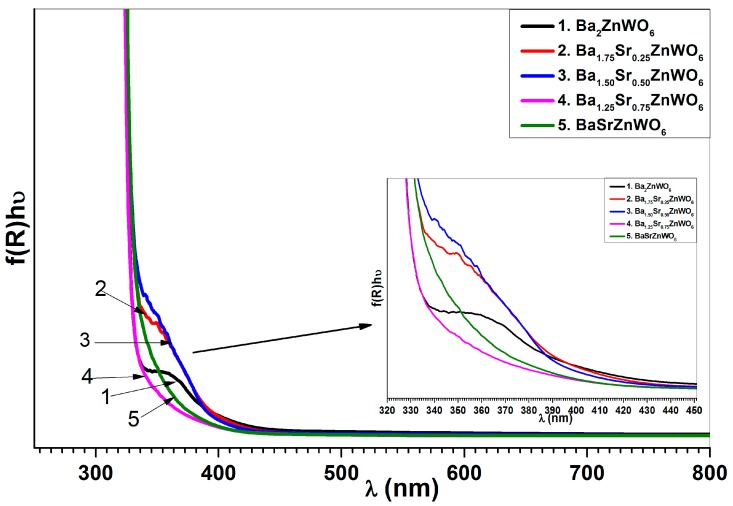
Absorbance versus the wavelength of Ba_2−*x*_Sr*_x_*ZnWO_6_ series.

**Figure 8:**
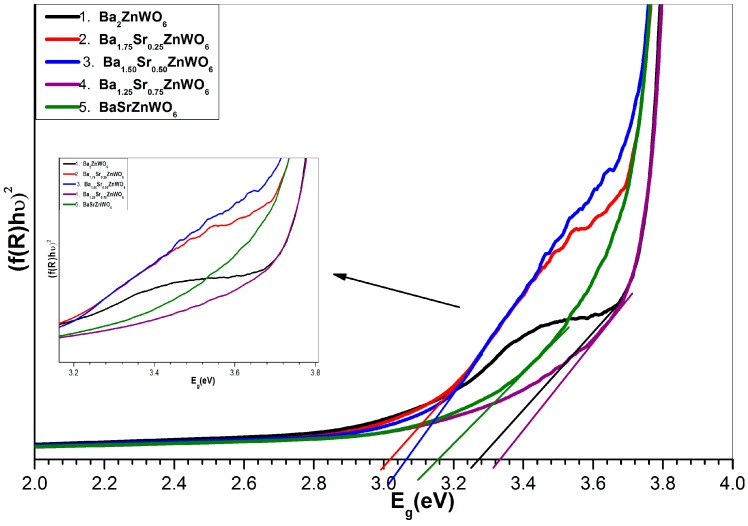
Indirect band gap plot for Ba_2−*x*_Sr*_x_*ZnWO_6_ (*x* = 0.00, 0.25, 0.5, 0.75, and 1.00) series.

The absorption coefficient can be calculated for the Ba_2−*x*_Sr*_x_*ZnWO_6_ series from the diffuse reflectance data using the Kubelka–Munk function [25]:

(3) $$F\left(R_{\infty }\right)=\frac{\alpha }{s}=\frac{{\left(1-R\right)}^{2}}{2R}$$

where $F\left(R_{\infty }\right)$ is the KM function, *α* is the absorption coefficient, *s* is the scattering coefficient, and *R* is the reflection coefficient. The absorbance (*f*(*R*)*hυ*) in relation to wavelength is shown in Figure 7. The absorbance can be used to observe the absorption edge for the samples that have values of 380, 410, 405, 370, and 389 nm for Ba_2_ZnWO_6_, Ba_1.75_Sr_0.25_ZnWO_6_, Ba_1.5_Sr_5_ZnWO_6_, Ba_1.25_Sr_0.75_ZnWO_6_, and BaSrZnWO_6_, respectively. The band gap energy of the series samples was calculated from the absorption edge [19] according to the relationship *E_g_* = 1240/*λ* (*λ* is the absorption edge wavelength and *E_g_* is the band gap [26]). In addition, the band gap energy was calculated for the samples using the Tauc plot [18], as shown in Figure 8, according to Equation (4).

(4) $${\left(F\left(R_{\infty })h\nu \right)\right)}^{n}=A\left(h\nu -E_{g}\right)$$

where hν is the incident photon energy, *A* is a proportional constant, $E_{g}$ is the band gap energy, and *n* takes values of 2 or 0.5 for indirect and direct transitions, respectively. Table 3 illustrates the bang gap energy according to the absorption edge and Tauc plot. The results of UV–vis diffuse reflectance and the optical energy gap of the sample series indicate that they can be classified as semiconductor materials [19,27]. 

In the case of complete substitution of Ba^2+^ with Sr^2+^, the material of Sr_2_ZnWO_6_ has a monoclinic (P2_1_/*n*) crystal structure [28,29] with 3.8 eV band gap energy [29].

#### 2.2.4. Photoluminescence Spectroscopy

**Figure 9:**
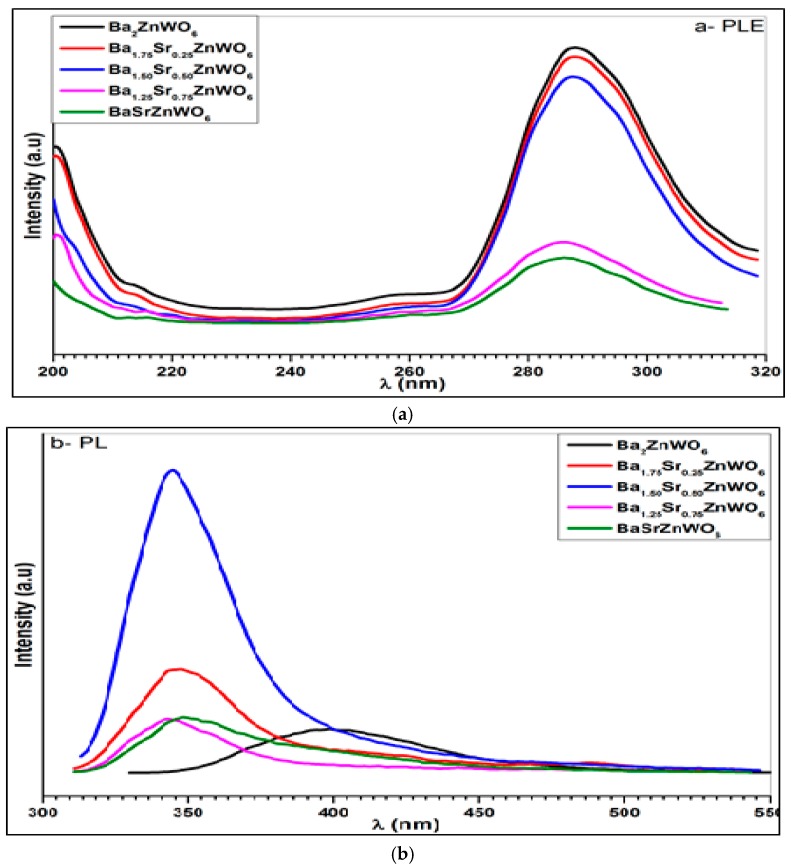
Photoluminescence excitation (**a**) and emission (**b**) spectrum of Ba_2−*x*_Sr*_x_*ZnWO_6_ series.

Figure 9 shows the excitation and photoluminescence emission spectra of the Ba_2−*x*_Sr*_x_*ZnWO_6_ (*x* = 0.00, 0.25, 0.50, 0.75, 1.00) double perovskite oxide series. The excitation spectra shown in Figure 9a were collected when *λ_em_* = 380 nm for Ba_2_ZnWO_6_, *λ_em_* = 346 nm for Ba_1.75_Sr_0.25_ZnWO_6_, *λ_em_* = 344 nm for Ba_1.5_Sr_0.5_ZnWO_6_, *λ_em_* = 343 nm for Ba_1.25_Sr_0.75_ZnWO_6_, and *λ_em_* = 349 nm for BaSrZnWO_6_. A broad band was observed between 260 and 320 nm, resulting from the electronic excitation of the O (2p) orbital-W (5d) orbital in octahedral WO_6_ [14,28]. In addition, the excitation peaks of the samples decrease with the increase in the Ba^2+^ ratio substitution of Sr^2+^ cations. The photoluminescence emission of the samples was investigated at *λ_ex_* = 290 nm for Ba_2_ZnWO_6_, *λ_ex_* = 288 nm for Ba_1.75_Sr_0.25_ZnWO_6_, *λ_ex_* = 287 nm for Ba_1.5_Sr_0.5_ZnWO_6_, and *λ_ex_* = 386 nm for Ba_1.25_Sr_0.75_ZnWO_6_ and BaSrZnWO_6_ displayed a spectral emission spread between 320 and 450 nm. Bugaris et al. [24] found a complimentary result where the emission peak of Ba_2_ZnWO_6_ displays a maximum at 539 nm when λ*_ex_* = 380 nm. In addition, there is a decrease in excitation intensity peaks with an increase in substitution. The photoluminescence (PL) of Ba_2_ZnWO_6_ has an emission spectrum peak at 380 nm and a FWHM of 70 nm. Similarly, Ba_1.75_Sr_0.25_ZnWO_6_, Ba_1.5_Sr_0.5_ZnWO_6_, Ba_1.25_Sr_0.75_ZnWO_6_, and BaSrZnWO_6_ have peaks at 345, 344, 343, and 342 nm with FWHMs of 40, 40, 40, and 50 nm, respectively. From the peaks of photoluminescence emission, the indirect band gap energy was calculated using the *E* = *hc*/*λ* equation for the series that was found to be 3.26, 3.5, 3.6, 3.6 and 3.65 eV for Ba_2_ZnWO_6_ Ba_1.75_Sr_0.25_ZnWO_6_, Ba_1.5_Sr_0.5_ZnWO_6_, Ba_1.25_Sr_0.75_ZnWO_6_, and BaSrZnWO_6_, respectively [30].

## 3. Materials and Methods

### 3.1. Samples Preparation

Ba_2−_*_x_*Sr*_x_*ZnWO_6_ (where *x* = 0.00, 0.25, 0.50, 0.75, 1.00) was synthesized using the solid-state interaction method from BaCO_3_ (barium carbonite), WO_3_ (tungsten trioxide), ZnO (zinc oxide), and NiO (nickel oxide) powders mixed in stoichiometric proportions according to the following chemical equation.

(5) $$\left(2-x\right){\mathrm{BaCO}}_{3}+x{\mathrm{SrCO}}_{3}+\mathrm{ZnO}+{\mathrm{WO}}_{3}\to {\;\mathrm{Ba}}_{2-x}{\mathrm{Sr}}_{x}{\mathrm{ZnWO}}_{6}+2{\mathrm{CO}}_{2}↑$$

Initially, the materials were used as purchased from Alfa Acer with a purity of 99.99%. Several different treatments of the samples were conducted to achieve the crystal structure. The raw materials were mixed and ground in an agate mortar with acetone, kept in crucibles, and subsequently heated in air at 800 °C for 12 h twice. The sample pellets were prepared in a round shape and heated at 1000 °C twice, following which the same procedure was repeated at 1200 °C. Between the steps for the heating treatment, the sample was ground for 2 h with acetone to increase the homogeneity of the sample at a rate of 10 °C per minute during the heating and cooling process.

### 3.2. Sample Characterization 

A Jeol JSM-6360 (JEOL Inc. Peabody, MA, USA) and high-resolution Stereo Scan LEO 440 SEM (LEO, Austin, TX, USA) were used to investigate the morphology and determine the homogeneity of the samples, as well as to obtain crystal-scale crystallization. At room temperature, the XRD data were recorded with a Bruker: D8 Advance (Bruker-Axs, Madison, WI, USA) using CuKα radiation (*λ* = 1.5406 Å) with a nickel filter. At 40 kV and 40 mA, data were collected for 2*θ* at 0.02-step sizes and 5-s count times in the 20°–80° range. The XRD data were analyzed using the Rietveld refinement method with the FullProf Suite program [31]. The crystalline size (*D*) [32] was calculated using the Debi–Scherer equation for all samples. At room temperature, the transmittance mode was investigated for all samples using the Satellite FTIR 5000 (ARCoptix S.A, Neuchatel, Switzerland) (with a wavelength range of 400–4000 cm^−1^) [33], where the important bands and peaks of the perovskite structure can be assigned. Using FTIR spectroscopy collected using the KBr pellet method, the material was mixed with KBr at 1:100 ratios for the FTIR measurement in the range of 400–2000 cm^−1^. The Raman spectra were collected in Raman HR (Stellarnet Inc., Tampa, FL, USA), using the high resolution Raman spectrometer with a range of 200–2200 cm^−1^ at 785 nm with a resolution of 4 cm^−1^. The Raman probe attaches to the laser via FC/APC and the spectrometer via SMA 905, and has integrated Raman filters and optics with a working distance to the sample of 4.5 mm, configured for the 785 nm laser. A UV–Vis spectrophotometer (UV-2550, Shimadzu, Chiyoda-ku, Tokyo) using BaSO_4_ as a reference is used to calculate the UV–Vis diffuse reflectance spectrum at room temperature. In addition, the UV–Vis reflectance spectrum is converted to absorbance using the Kubella–Munk method to estimate the edge of absorption and band gap of the Ba_2−*x*_Sr*_x_*ZnWO_6_ double perovskite powder series. A Perkin Elmer LS55 fluorescence spectrometer (Perkin-Elmer, Wokingham, UK) was used to investigate the emission and excitation of the Ba_2−*x*_Sr*_x_*ZnWO_6_ double perovskite series at room temperature.

## 4. Conclusions

The Ba_2−*x*_Sr*_x_*ZnWO_6_ (*x* = 0.00, 0.25, 0.50, 0.75, 1.00) double perovskite series was prepared by the solid-state reaction technique. In addition, techniques such as X-ray diffraction, scanning electronic microscopy, Fourier transform infrared spectra, Raman spectra, UV-Vis diffuse reflectance, photoluminescence excitation, and emission spectra were investigated. SEM revealed that the prepared Ba_2−*x*_Sr*_x_*ZnWO_6_ samples crystallized at micrometer scales, where the crystal structure of the samples was determined by XRD as a cubic Fm-3m space group. The lattice parameter decreased with an increase in the proportion of substitution from *x* = 0 to 1, where the result of FTIR also confirmed the double perovskite structure. The Raman spectra of W–O bonds indicated a systematic decrease and re-shifting with an increasing Ba substitution. Strong UV-Vis absorption was found between 350 and 410 nm and smaller optical bandgap energy, 3.02 eV, was found for Ba_1.75_Sr_0.25_ZnWO_6_ compared to the Sr-free sample. The excitation photoluminescence spectra displayed broad bands between 260 and 320 nm, which were assigned to the charge transfer band of Ba_2−*x*_Sr*_x_*ZnWO_6_. Ba_2−*x*_Sr*_x_*ZnWO_6_ displays photoluminescence in the near-UV and visible region. This feature makes Ba_2−*x*_Sr*_x_*ZnWO_6_ a potential semiconductor in optoelectronics applications.

## Figures and Tables

**Table 1:** Atom coordinates of Ba_2−*x*_Sr*_x_*ZnWO_6_ (*x* = 0.00, 0.25, 0.50, 0.75, 1.00) double perovskite series following Rietveld refinement of X-ray powder diffraction.

Cation/Anion	Coordinates	Ba_2_ZnWO_6_	Ba_1.75_Sr_0.25_ZnWO_6_	Ba_1.5_Sr_0.5_ZnWO_6_	Ba_1.25_Sr_0.75_ZnWO_6_	BaSrZnWO_6_
Ba^+2^	X	0.2500	0.2500	0.2500	0.2500	0.2500
	Y	0.2500	0.2500	0.2500	0.2500	0.2500
	Z	0.2500	0.2500	0.2500	0.2500	0.2500
Sr^+2^		---------	0.2500	0.2500	0.2500	0.2500
		---------	0.2500	0.2500	0.2500	0.2500
		---------	0.2500	0.2500	0.2500	0.2500
Zn^+2^	X	0.500	0.500	0.500	0.500	0.500
	Y	0.500	0.500	0.500	0.500	0.500
	Z	0.500	0.500	0.500	0.500	0.500
W^+6^	X	0.00	0.00	0.00	0.00	0.00
	Y	0.00	0.00	0.00	0.00	0.00
	Z	0.00	0.00	0.00	0.00	0.00
O_1_^−2^/O_2_^−2^	X	0.23990	0.25815	0.24414	0.22556	0.24414
	Y	0	0	0	0	0
	Z	0	0	0	0	0

**Table 2:** The tolerance factor and the parameter of crystal structure of Ba_2−*x*_Sr*_x_*ZnWO_6_ (*x* = 0.00, 0.25, 0.50, 0.75, 1.00) following Rietveld method refinement.

Empirical Formula	Ba_2_ZnWO_6_	Ba_1.75_Sr_0.25_ZnWO_6_	Ba_1.5_Sr_0.5_ZnWO_6_	Ba_1.25_Sr_0.75_ZnWO_6_	BaSrZnWO_6_
Space group	Fm-3m	Fm-3m	Fm-3m	Fm-3m	Fm-3m
*α* (Å)	8.11834	8.100679	8.076869	8.060842	8.039361
*α*/*β*/*γ*	90	90	90	90	90
*V* (Å^3^)	535.0590	531.57465	526.9001	523.7707	519.5751
*D* (nm)	105.09	78.38	47.41	88.03	84.59
*T*	1.007	1.00	0.992	0.990	0.998
*R_WP_*	9.78	9.71	8.87	13.6	11.3
*R_P_*	8.75	10.2	8.15	10.3	10.8
$\chi^{2}$	1.7738	1.886	1.681	2.813	2.356

**Table 3:** Illustration of band gap energy according to the absorption edge and Tauc plot for direct transition.

Bang Gap Energy	Ba_2_ZnWO_6_	Ba_1.75_Sr_0.25_ZnWO_6_	Ba_1.5_Sr_0.5_ZnWO_6_	Ba_1.25_Sr_0.75_ZnWO_6_	BaSrZnWO_6_
Cut-off wavelength (nm)	380	410	405	370	389
*E_g_* (eV) by cutoff wavelength	3.26	3.02	3.06	3.35	3.18
*E_g_* (eV) by Tauc plot	3.27	3.02	3.07	3.34	3.16
